# Sca-1 identifies a trophoblast population with multipotent potential in the mid-gestation mouse placenta

**DOI:** 10.1038/s41598-017-06008-2

**Published:** 2017-07-17

**Authors:** Bryony V. Natale, Christina Schweitzer, Martha Hughes, Maria A. Globisch, Ramie Kotadia, Emilie Tremblay, Priscilla Vu, James C. Cross, David R. C. Natale

**Affiliations:** 1 Department of Comparative Biology and Experimental Medicine, Faculty of Veterinary Medicine, University of Calgary, Calgary, AB T2N4N1 Canada; 2 Department of Reproductive Medicine, Faculty of Medicine, University of California San Diego, La Jolla, CA 92093 USA

## Abstract

Trophoblast stem (TS) cells in the mouse derive from the polar trophectoderm of the blastocyst and persist through early gestation (to E8.5) to support placental development. Further development and growth is proposed to rely on layer-restricted progenitor cells. Stem cell antigen (Sca) -1 is a member of the Ly6 gene family and a known marker of stem cells in both hematopoietic and non-hematopoietic mouse tissues. Having identified that *Sca-1* mRNA was highly expressed in mouse TS cells in culture, we found that it was also expressed in a subset of trophoblast within the chorion and labyrinth layer of the mouse placenta. Isolation and *in vitro* culture of Sca-1^+^ trophoblast cells from both differentiated TS cell cultures and dissected mouse placentae resulted in proliferating colonies that expressed known markers of TS cells. Furthermore, these cells could be stimulated to differentiate and expressed markers of both junctional zone and labyrinth trophoblast subtypes in a manner comparable to established mouse TS cell lines. Our results suggest that we have identified a subpopulation of TS cell-like cells that persist in the mid- to late- gestation mouse placenta as well as a cell surface protein that can be used to identify and isolate these cells.

## Introduction

Progress has been made in reproductive medicine in many areas. However, for placenta-related pathologies the etiology and mechanisms underlying pregnancy-related diseases are not understood. A poorly functioning placenta is a contributing cause of several of these, including **i**ntra**u**terine **g**rowth **r**estriction (IUGR) and preeclampsia. Despite decades of research investigating pregnancy and fetal outcome, there is no true understanding of how the basic biological processes involved in placental development fail and in many cases physicians can only manage the mothers’ symptoms. Identification of a human **t**rophoblast **s**tem (TS) cell might provide the potential for regenerative medicine to treat placental pathologies. TS cells in the mouse placenta are thought to be depleted by **e**mbryonic day (E)8.5^1^. However, in other organs tissue-specific stem/progenitor cells provide a reservoir of undifferentiated cells supporting the proliferation and differentiation required for adaptation to stress and/or injury^2, 3^. Following this line of reasoning, we sought to identify a subpopulation of trophoblast cells that persisted beyond mid-gestation that might have multipotent, proliferative potential.

The placenta is the first organ to form during development and its principle function is to facilitate the exchange of nutrients and waste, while providing immune protection and production of hormones that adapt maternal physiology to the developing pregnancy. While there are some differences in the structure and cell types between the mouse and human placenta, both are hemochorial, have invasion of trophoblast cells into the uterine wall, and share the basic functions and gene expression underlying their development^4, 5^. The availability of genetic tools and the strong correlation with the human placenta makes the mouse an ideal model in which to investigate TS cells^6, 7^.

The mouse placenta is composed of three layers: the maternal decidua, the junctional zone and the labyrinth. Each contains distinct populations of terminally differentiated trophoblast, some that remain localized, and others that migrate. The decidua, the outermost layer, is primarily composed of maternally derived cell types, but is home to the fetal-derived **sp**iral **a**rtery **t**rophoblast **g**iant **c**ell (SpA-TGC). Separating the decidua from the mid-layer junctional zone are **p**arietal **t**rophoblast **g**iant **c**ells (P-TGC). The junctional zone is made up of spongiotrophoblast and glycogen trophoblast cells; the latter begin to store glycogen near mid-gestation^6, 7^, followed by a migration to the decidua^8^. The labyrinth, which is closest to the fetus, is a complex villous structure that is formed through the branching morphogenesis of trophoblast cells from the chorion^9, 10^, is bathed in maternal blood and presents a large surface area for nutrient exchange. Within the labyrinth lies a network of fetal vessels, which connect to the umbilical cord. Four cellular layers separate the maternal and fetal blood spaces. **S**inusoidal **t**rophoblast **g**iant **c**ells (S-TGC) line maternal blood spaces followed by two layers of **syn**cytio**t**rophoblast (SynT1 and SynT2) cells, and then the fetal endothelial cell layer that lines the fetal blood spaces. The mature placenta, while formed by E10.5, continues to grow until E16.5. Any disruption to the layers or the differentiated sub-types has the potential to cause pregnancy related complications^7^.

As human TS cells have not been definitively identified in the human placenta, and their characterization might offer future treatment of placental pathologies, we use the mouse to identify factors that promote and/or recruit TS and progenitor populations in hope to facilitate further understanding of human trophoblast stem- and progenitor cells. *Cdx2* is one of the first genes detected in cells differentiating to the trophoblast lineage^11, 12^. The mouse trophoblast lineage is reported to be irreversibly committed at the blastocyst stage, though it has been shown that at the 16-cell morulae stage, *Cdx2*-positive outer cells can contribute to the inner cell mass if they are moved to an internal position and likewise inner cells repositioned to the outer surface can form trophectoderm^13^, reminding us of the delicate balance between pluripotent and lineage restricted precursors. Mouse TS cells are derived from the polar trophectoderm of the blastocyst and following implantation, proliferate rapidly to support placental development. Early in gestation, TS cells give rise to: 1) the ectoplacental cone from where the junctional zone trophoblast are derived, and 2) the **ex**traembryonic **e**ctoderm (ExE) which gives rise to the labyrinth trophoblast. The ectoplacental cone does not retain TS cells, rather it maintains a population of *Ascl2* ^+^ trophoblast progenitor cells, which differentiate and form the junctional zone^14–16^. Lineage tracing experiments have shown that *Tpbpa* ^+^ trophoblast progenitors within the ectoplacental cone give rise to spongiotrophoblast, glycogen trophoblast as well as P-TGC and SpA-TGC s^14^. A specific subset of these progenitor cells express Blimp1 and only differentiate to glycogen trophoblast, SpA-TGC and canal TGCs^15^. In contrast, the ExE does maintain a population of TS cells (*Esrrb* ^+^, *Eomes* ^+^), which give rise to the chorion from where labyrinth progenitors (Epcam^+^) and the differentiated trophoblast cells of the labyrinth are derived^1, 14, 17, 18^. These TS cells are mostly depleted by E8.5, with the few remaining, present in the chorion^1^. It has been proposed that Epcam^+^ trophoblast progenitor cells within the chorion-derived labyrinth persist until E14.5^18^, and when these cells differentiate, they become the SynT and S-TGC s of the labyrinth layer. Established mouse TS cell lines, isolated from blastocysts, have been used as an investigational tool *in vitro*, to understand the molecular mechanisms supporting proliferation and differentiation, thereby contributing to the current understanding of placental development and function.

In the human placenta, the proliferating cyto-trophoblast cells found in the villi underlying the SynT cells, contribute to villous development in a similar manner to Epcam^+^ trophoblast in the mouse^19, 20^. These proliferating cyto-trophoblast cells are abundant in early pregnancy, however their population declines through gestation^19^. These cells are considered progenitors and they contribute to the multi-nucleated syncytial layer. In studies of various human pregnancy related conditions, rates of cyto-trophoblast proliferation are altered^21^, suggesting that *in utero* factors can affect trophoblast proliferation. As such, identifying whether the placenta maintains a stem and/or a stem cell-like progenitor population (or whether trophoblast cells maintain some plasticity), and a means to identify and isolate these cells would provide a means to understand the *in vivo* signals that control their proliferation and subsequent differentiation. Localization and isolation of human TS cells has been a challenge and there is not currently a good cell surface marker with which to identify them.

In a mouse study to identify hematopoietic stem cells in the placenta, EGFP expression under the control of the cell surface protein, **s**tem **c**ell **a**ntigen-**1** (Sca-1, also known as Ly6A) promoter, was described in the trophoblast cells of the ExE in early gestation and cells of non-hematopoietic lineage in the labyrinth layer after mid-gestation^22^. Recently, in work by Tsukiyama *et al*.^23^, *Sca-1* was identified in microarray data from TS cell cultures derived and grown in improved, defined conditions^23^. Sca-1 expression has been a useful surface marker of stem/progenitor cells in other tissues thereby leading us to ask whether Sca-1 would make a good cell surface marker of proliferative multipotent trophoblast cells in the mouse placenta. The goals of the current study were to: 1) Assess whether the cell surface marker Sca-1 could be used to identify a population of proliferative multipotent trophoblast cells, 2) Identify whether a persistent trophoblast population with multipotent potential could be isolated from the mid-gestation mouse placenta.

## Results

### Identification of Sca-1^+^ Trophoblast Cells

**Figure 1:**
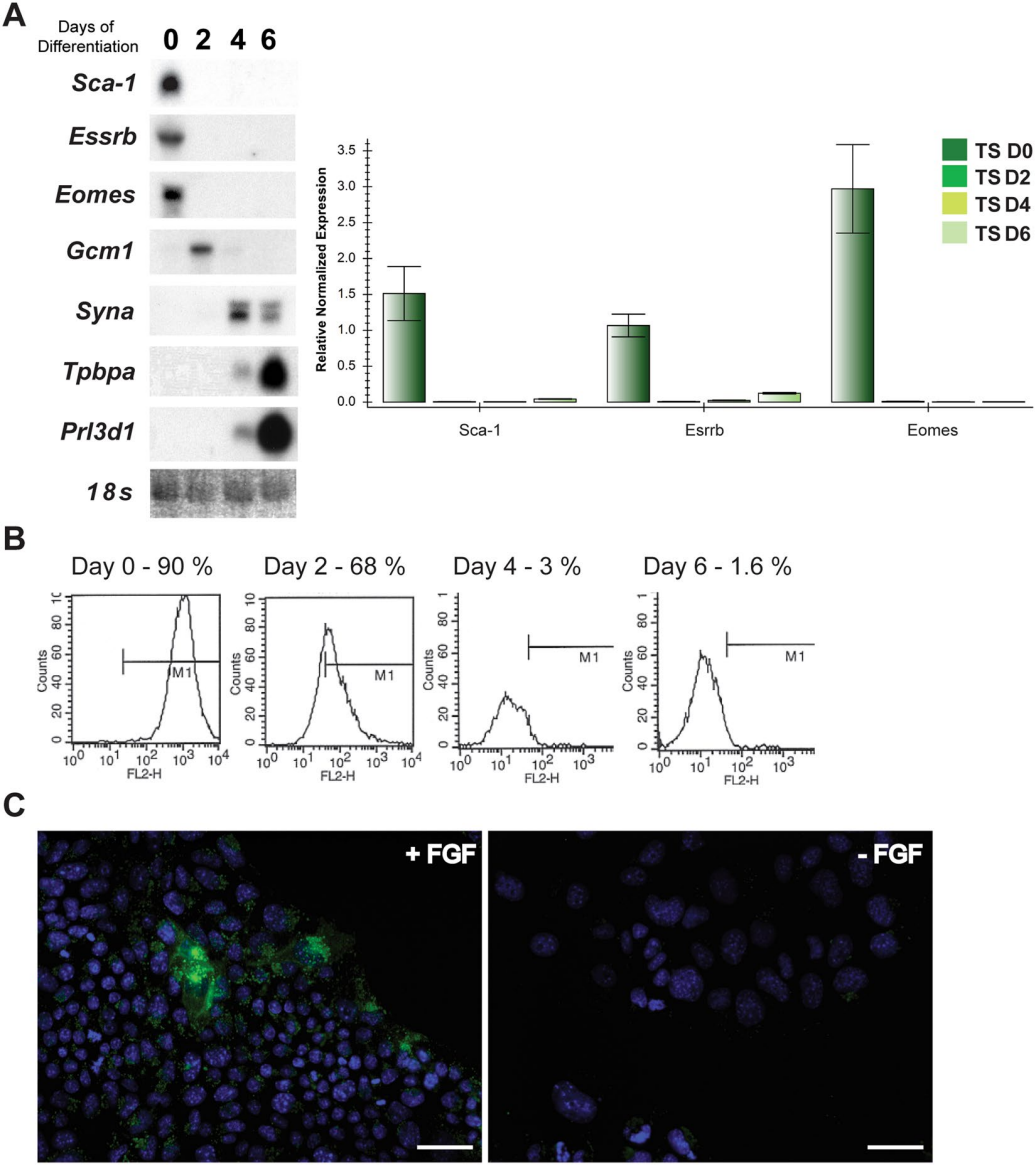
Sca-1 was determined to be highly expressed in undifferentiated TS cells in culture by Northern blot and qRT-PCR (**A**) and FACS analysis (**B**). Day 0/0 represents undifferentiated, proliferating TS cells while 2, 4, 6 and Day 2, Day 4 and Day 6 represent cultures differentiated for 2, 4 or 6 days. Sca-1 was also expressed in TS cells in culture as detected by immunofluorescence (**C**; +FGF) however became undetectable following differentiation (**C**; -FGF). Immunofluorescence in cells (**C**) in the presence and absence of FGF matched the pattern of expression observed by FACS (**B**), however was not obvious in as a high frequency (+FGF) which is likely due to the more sensitive detection of Sca-1 expression by FACS. Scale bar = 100 um. Northern blot results shown in (**A**) are cropped and organized to be presented as a composite from multiple blots/films.

*Sca-1* was found to be highly expressed in established, proliferating TS cell cultures, as shown by mRNA analysis by Northern blot and qRT-PCR. *Sca-1* expression was greatest in undifferentiated TS cells, while in differentiating cells, it was rapidly down-regulated (Fig. 1), in a pattern similar to the TS cell markers *Essrb* ^24^, *Cdx2* ^12^ and *Eomes* ^25^ (Fig. 1A). After 6 days of differentiation neither the TS cell markers nor *Sca-1* were detectable by Northern blot and were significantly down-regulated in qRT-PCR assays. Sca-1 protein expression was confirmed using FACS analysis, and the protein expression pattern complimented the mRNA pattern (Fig. 1B,C), with greatest expression in undifferentiated cells. Of significance, after six days of differentiation, a small subpopulation of cells (<2%) remained Sca-1 positive among the differentiated trophoblast.

### Sca-1 as a cell surface marker of colony forming trophoblast cells

**Figure 2:**
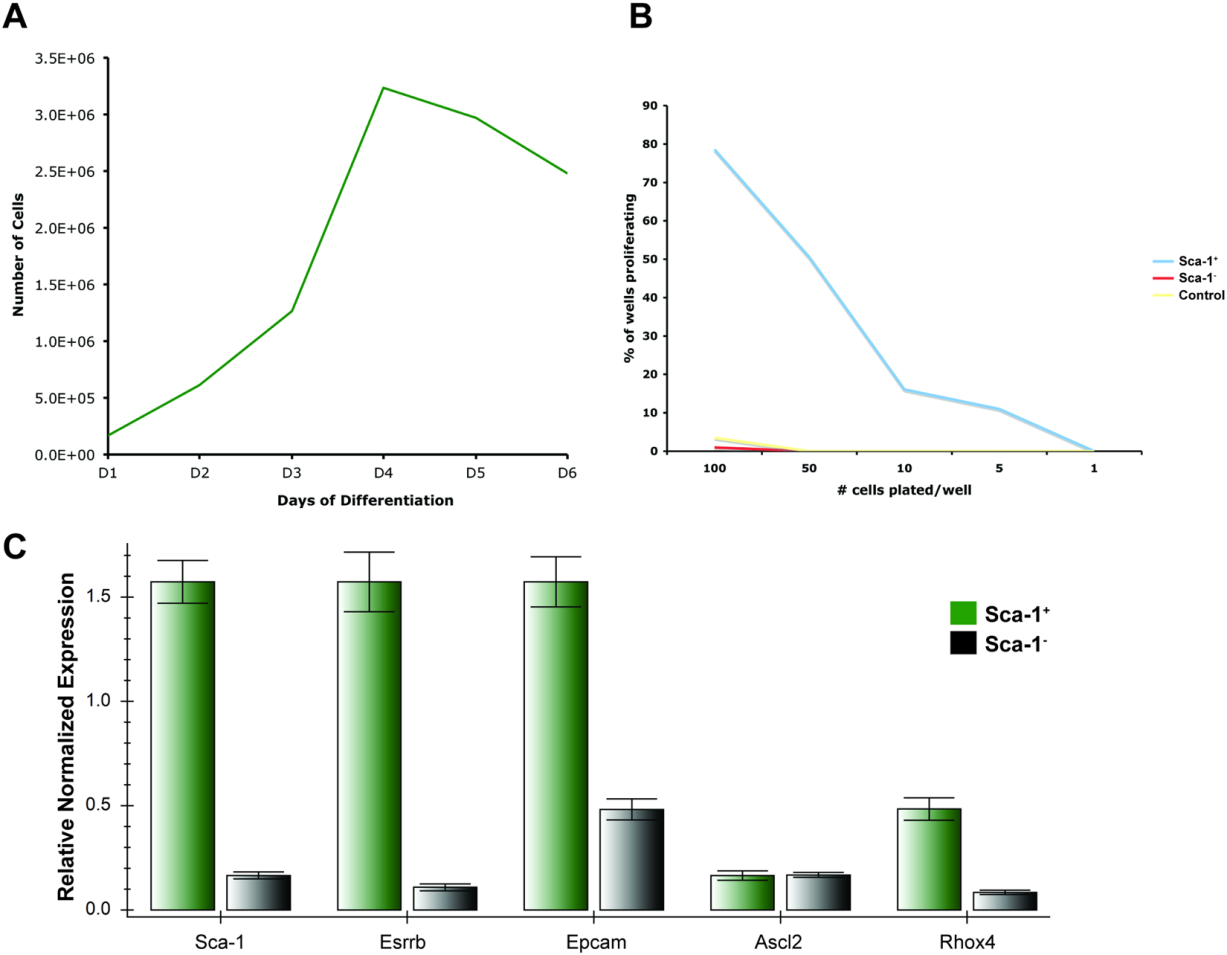
Upon removal of FGF4, mouse TS cell cultures were initially proliferative but increased in number only until day 4 of differentiation (**A**). Sca-1^+^ trophoblast cells isolated from non-proliferating, differentiated TS cultures and plated at decreasing densities in conditions to support TS proliferation formed colonies however Sca-1^−^ and non-sorted, differentiated TS cells did not (**B**). When these cells were compared to the Sca-1^−^ subpopulation of trophoblast cells by qRT-PCR, Sca-1^+^ positive cells displayed higher expression of *Sca-1* and the trophoblast stem and progenitor markers, *Esrrb* and *Epcam, Rhox4*, respectively, while *Ascl2* was not different (**C**).

TS cells, in culture, stop proliferating and undergo changes in morphology and gene expression as they terminally differentiate^26, 27^. After four to six days of differentiation, our TS cells were no longer proliferative and stopped increasing in number (Fig. 2A). Considering the subpopulation (~1.6%) of Sca-1^+^ cells within the 6-day differentiated TS cell culture, and its use as a cell surface marker in other tissue-specific stem cells, we hypothesized that Sca-1 expression may identify an undifferentiated trophoblast population. To test this, we conducted a series of *in vitro* culture and FACS experiments to isolate the Sca-1^+^ population from the differentiated TS cell cultures.

**Figure 3:**
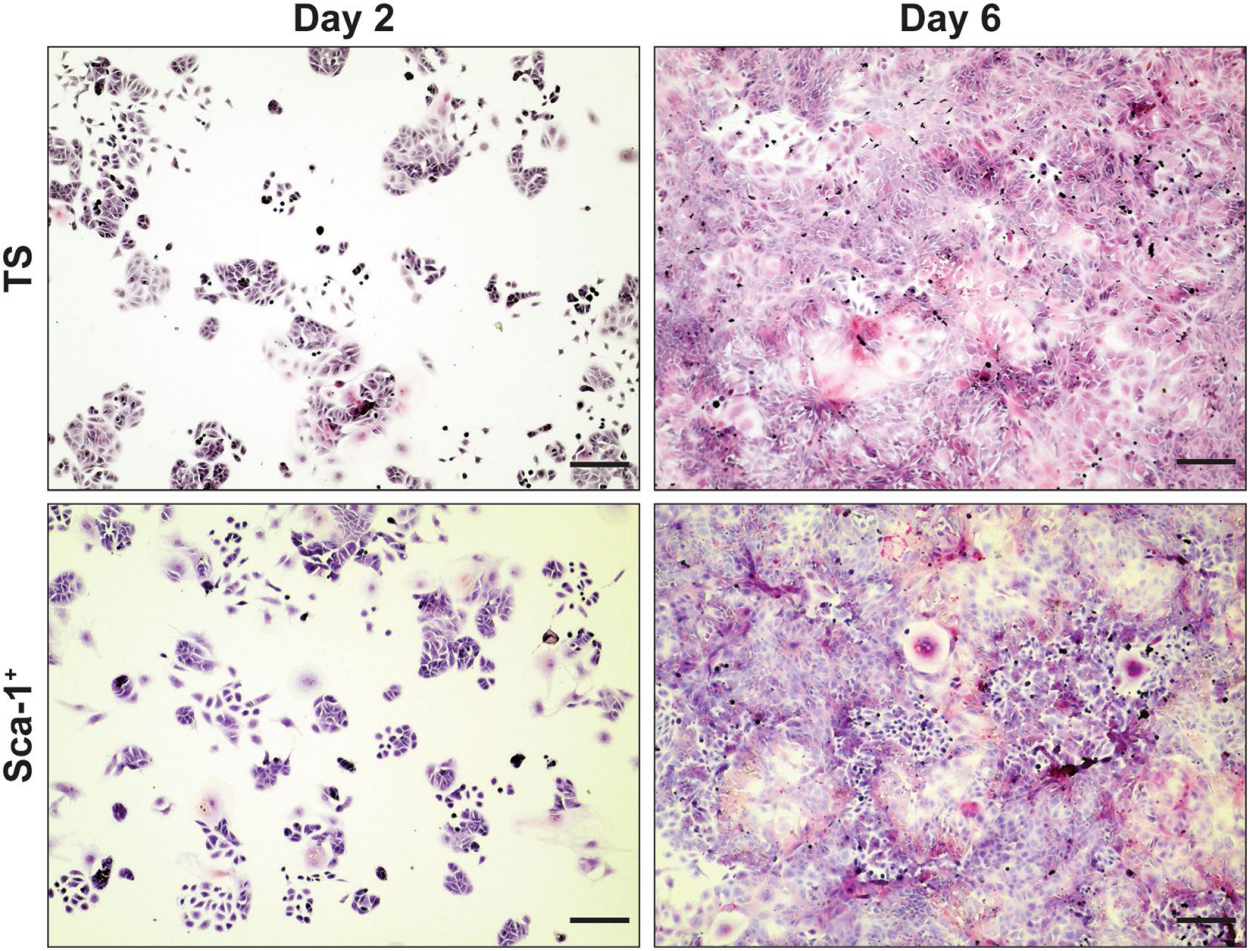
Sca-1^+^ trophoblast isolated from differentiated TS cell cultures and re-plated in TS media plus growth factors formed colonies comparable to TS cells plated at the same density from proliferating cultures (compare TS and Sca-1^+^, Day 2). After six days of culture in TS media plus growth factors, both Sca-1^+^ and TS cells were confluent and appeared similar in morphology (compare TS and Sca-1^+^, Day 6). Cells were stained with hematoxylin and eosin. Scale bar = 500 um.

**Figure 4:**
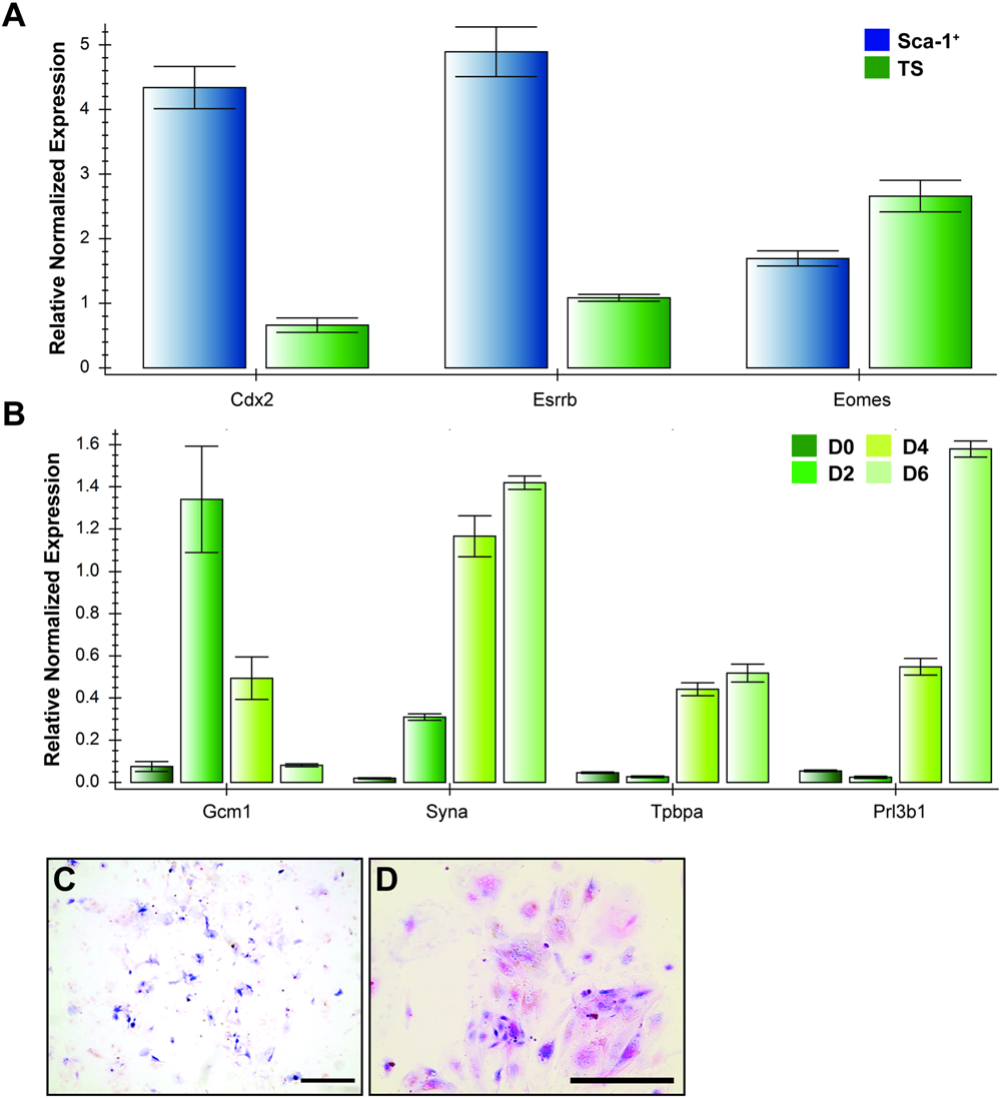
TS cell markers, *Cdx2* and *Esrrb* were more highly expressed in Sca-1^+^ trophoblast isolated from differentiated TS cell cultures than in non-sorted, proliferating TS cell cultures (**A**). Sca-1^+^ trophoblast isolated from differentiated TS cultures gave rise to proliferating trophoblast cell lines that when differentiated by the removal of growth factors, expressed genes indicative of syncytiotrophoblast (*Gcm1, Syna*), spongiotrophoblast (*Tpbpa*) and TGCs (*Prl3b1*) (**B**). These differentiated cells were also positive for alkaline phosphatase activity (**C**, blue) and PAS (**D**, magenta/arrowhead) indicating differentiation of sinusoidal TGCs and glycogen trophoblast, respectively. D0 represents undifferentiated, proliferating TS cells while, D2, D4, D6 represent cultures differentiated for 2, 4 or 6 days. In C, scale bar = 500 um. In D, scale bar = 100 um.

TS cells were differentiated for four days and the Sca-1^+^ and Sca-1^−^ subpopulations were isolated (by FACS or MACS) and re-plated in media containing growth factors to support colony formation. We reasoned that if Sca-1^+^ cells were undifferentiated trophoblast cells, then under permissive conditions they would proliferate and form colonies. Undifferentiated TS cell cultures, although proliferative, contain cells in varying states of differentiation^27^. While FGF4 and other factors keep TS cell cultures in their most proliferative, least differentiated state^26, 27^, these cells are not cell cycle synchronized and cannot be without causing differentiation. As such not every cell in the population may have colony-forming potential. Therefore, we used a colony-forming assay^28^ to determine what percentage of cells from differentiated trophoblast cultures, had colony forming, proliferative ability. We found that proliferating TS cells formed colonies at a frequency of 1/15. However, Sca-1^+^ trophoblast cells isolated from differentiated cultures had colony forming ability at a frequency of 1/42, while neither the differentiated TS cell culture nor the Sca-1^−^ population formed colonies, confirming our hypothesis that Sca-1^+^ trophoblast cells had the potential to form proliferative colonies (Fig. 2B). qRT-PCR assessment of the subpopulations showed that the Sca-1^+^ cells expressed *Esrrb* (TS cell marker), *Epcam* and *Rhox4* (markers of trophoblast progenitor cells) at a greater level than the Sca-1^**−**^ cells (Fig. 2C). Immediately after the sort, Sca-1^+^ cells grew more slowly than established, freshly passaged TS cell cultures, but after 2 days they caught up and at day 6, appeared to be similar to TS cells (Fig. 3). As proliferating TS cell cultures are heterogeneous with respect to stem and progenitor populations, we reasoned that isolation by Sca-1 expression could enrich for a population of trophoblast cells that are in a more homogeneous state of “stemness”. To test this, we performed a qRT-PCR assessment of published TS cell markers. Sca-1^+^ cells expressed significantly more *Cdx2 and Esrrb*, though less Eomes than proliferating TS cell cultures (Fig. 4A).

Sca-1^+^ cells isolated from differentiated TS cell populations, allowed to form colonies in proliferative conditions, were stimulated to differentiate by the removal of growth factors. The culture stopped proliferating and cell morphology changed, resembling that of differentiated TS cells including both sinusoidal TGCs as indicated by alkaline phosphatase activity (Fig. 4C, blue) as well as gly-T as indicated by PAS staining, (Fig. 4D, magenta). qRT-PCR analysis of the differentiated Sca-1^+^ sorted cells, showed that they expressed markers of many differentiated trophoblast subtypes, including *Gcm1, Syna, Tpbpa* and *Prl3b1* (Fig. 4B). This confirms that Sca-1^+^ trophoblast cells have the potential to become differentiated sub-types of both labyrinth and junctional zone layers, under permissive conditions.

### Sca-1^+^ and Epcam^+^ trophoblast are different subpopulations

**Figure 5:**
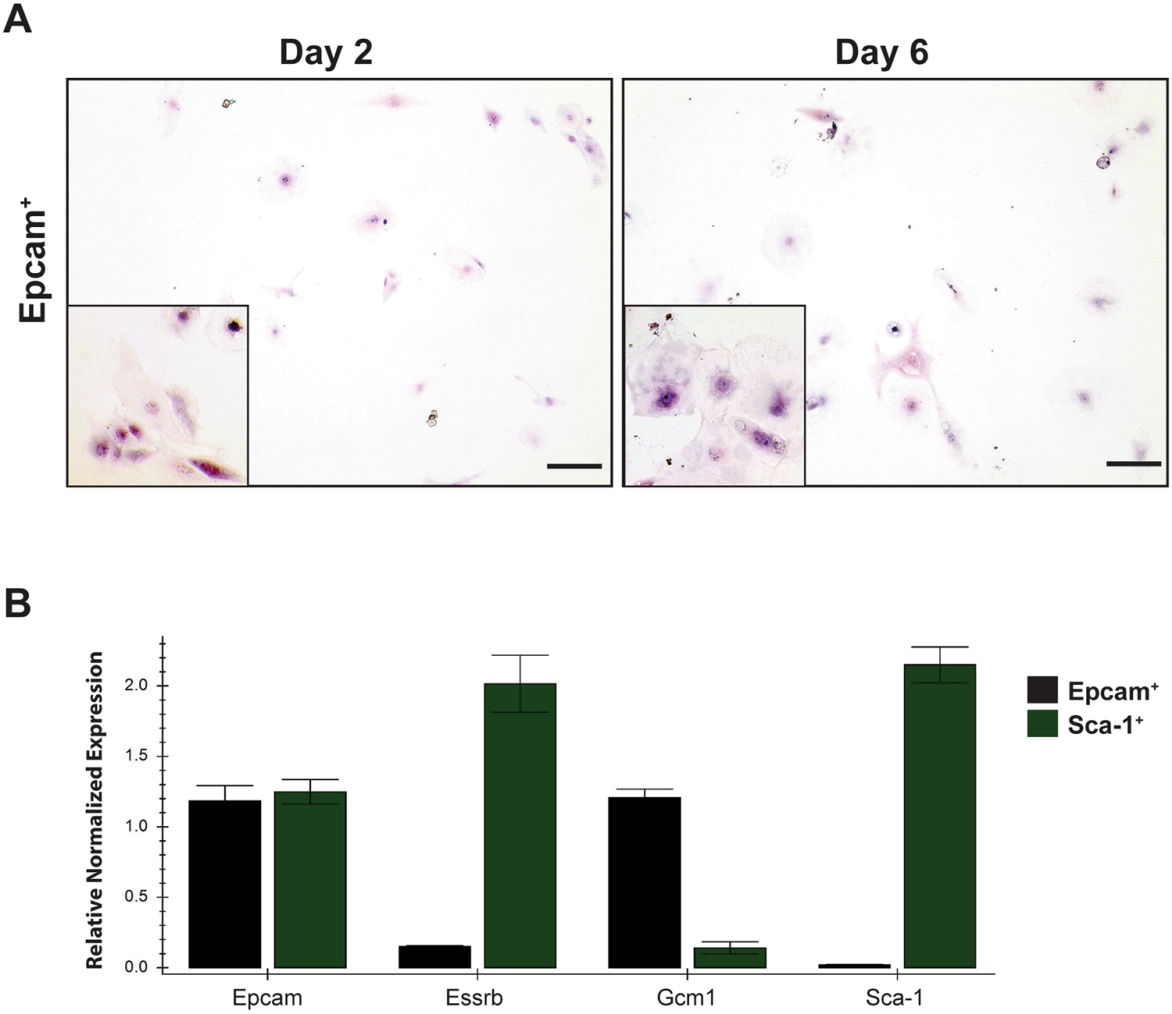
Epcam^+^ trophoblast isolated from TS cell cultures and re-plated in TS media containing growth factors to promote proliferation formed small colonies after 2 days of culture but did not proliferate (**A**, compare Day 2 to Day 6). After 6 days in culture in TS media plus growth factors, the cells had not expanded and had the morphology of differentiated trophoblast, with large nuclei and cell bodies (compare Day 6 to Fig. 3, Day 2). Sca-1^+^ cells compared to Epcam^+^ cells displayed increased expression of the TS cell marker, *Esrrb* while Epcam^+^ cells expressed the syncytiotrophoblast marker, *Gcm1* but not *Sca-1* or *Esrrb* (**B**). Insets in (**A**) show morphology of plated Epcam^+^ trophoblast cells at high magnification (400x). Cells were stained with hematoxylin and eosin. Scale bar = 500 um.

The labyrinth contains a proliferative subpopulation of Epcam^+^, labyrinth-restricted progenitor cells^18^. To test if Sca-1^+^ and Epcam^+^ cells were the same population, we isolated both Sca-1^+^ and Epcam^+^ cells from differentiated (four days) TS cell cultures, and re-plated both populations in proliferating conditions to compare their growth and proliferation potential. As with previous experiments, Sca-1^+^ cells formed colonies and reached 80% confluence every 3 days. Epcam^+^ cells, however, did not have the same growth and proliferation characteristics. Instead, Epcam^+^ cells formed very small colonies (<6 cells) after 2 days, but then stopped proliferating and began to differentiate; the cells could be cultured for 6 days, but could not be passaged or expanded (Fig. 5A). qRT-PCR comparison of sorted Sca-1^+^ and Epcam^+^ cells showed different gene expression patterns for markers of trophoblast proliferation and differentiation; Sca-1^+^ cells expressed *Epcam*, while Epcam^+^ cells did not appreciably express Sca-1. Furthermore, Sca-1^+^ cells preferentially expressed *Esrrb*, while Epcam^+^ cells preferentially expressed *Gcm1*, a marker of syncytiotrophoblast (Fig. 5B). Gene expression in Epcam^+^ cells was similar to that observed by Ueno *et al*.^18^ Sca-1^+^ and Epcam^+^ trophoblast subpopulations were indeed distinct, as within a differentiated TS cell population, Sca-1^+^ cells could be isolated and proliferated once returned to permissive conditions, whereas Epcam^+^ cells could not. Both Epcam^+^ and Sca1^+^ cells were isolated from culture after 1, 2 and 3 days of differentiation and the results were the same regardless of the time spent in the absence of FGF4.

### Identification and Isolation of Sca-1^+^ cells in the mid-gestation placenta

**Figure 6:**
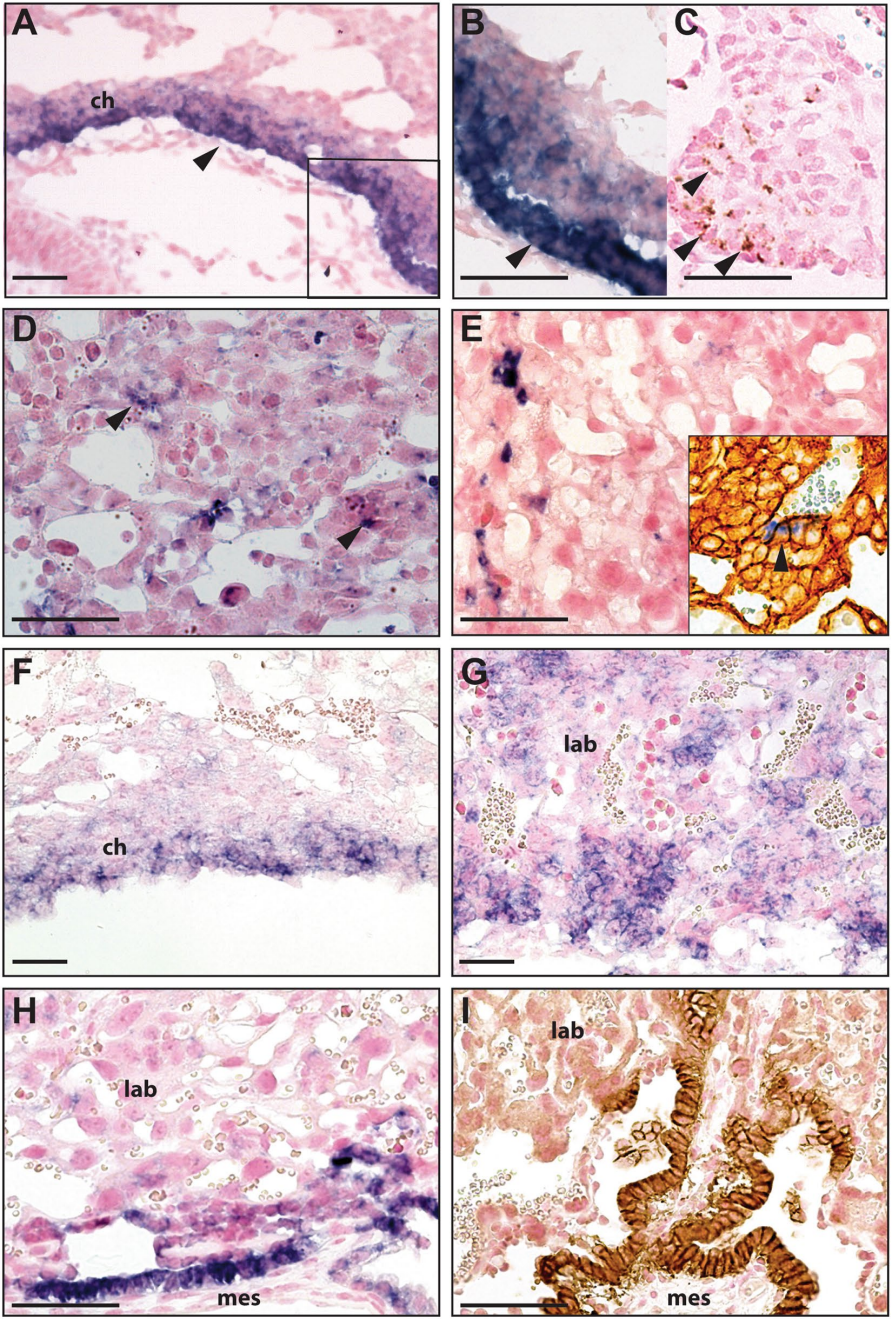
*Sca-1* was expressed in the chorion of the mouse placenta at E8.5 (**A**; arrow). The inset in (**A**) is shown at higher magnification in (**B**) and from a serial section in (**C**), where protein expression is indicated by arrows. Controls in which no primary antibody was used showed no positive staining (not shown). *Sca-1* was expressed in the labyrinth at E10.5 (**D**) and E16.5 (**E**). The inset in (**E**) shows *Sca-1* expression (arrowhead, blue; ISH) in cytokeratin positive trophoblast of the labyrinth (brown; IHC). *Epcam* mRNA expression was also detected in the chorion and appeared to overlap with *Sca-1* expression at E8.5 (**F**). At E10.5, *Epcam* expression was more broadly expressed in the labyrinth than *Sca-1* (**G**, compare with **D**) but by E16.5, both Epcam mRNA and protein were appreciably reduced however still present in more cells in the labyrinth than *Sca-1* (**H**,**I**, compare with **E**). ch, chorion; mes, embryonic mesoderm; lab, labyrinth. Scale bar = 100 um.

To confirm the presence of a Sca-1^+^ trophoblast cell population *in vivo*, histological assessment of placentae by $\underline{{\boldsymbol{i}}}n\,\underline{{\boldsymbol{s}}}itu$ ***h*** *ybridization* (ISH) was performed. It has been reported that *Sca-1* mRNA is expressed in the ExE and the chorion until E7.5^22^. As such, we conducted our analysis from E8.5 through E18.5. We observed *Sca-1* trophoblast expression in the chorion layer, closest to the fetal mesoderm and chorionic trophoblast interface at E8.5 (Fig. 6A,B, arrow; 6C, by IHC). At E10.5 and E12.5, *Sca-1* was expressed in trophoblast cells with a small compact morphology (Fig. 6D), suggestive of undifferentiated cells^29^. By E16.5, *Sca-1* expression was reduced and restricted to rare single cells within the labyrinth (Fig. 6E). This pattern remained consistent through E18.5. Although at most stages, *Sca-1* ^+^ cells could be identified as trophoblast by morphology and localization within the labyrinth layer, we also conducted *Sca-1* ISH/cytokeratin IHC and identified dual positive cells, confirming that *Sca-1* was expressed in labyrinth trophoblast (Fig. 6E, inset).

Epcam^+^ labyrinth-specific trophoblast progenitor cells reside in the chorion at E8.5 and persist in the labyrinth in small clusters until E14.5^18^. To determine whether *Sca-1* and *Epcam* overlapped we also examined *Epcam* expression. At E8.5, *Epcam* was restricted to trophoblast within the chorion (Fig. 6F). At E10.5, it was broadly expressed, but restricted to the labyrinth layer as reported^18^ and by E16.5 and 18.5, although *Epcam* expression was greatly reduced, it was still detectable in a single layer of cells adjacent to the trophoblast-mesoderm interface along the fetal side of the placenta (Fig. 6H; and I, by IHC). Based on these findings, *Sca-*1 and *Epcam* overlapped in trophoblast at E8.5 and E10.5 (compare Fig. 6A and F), but appeared to be distinct populations later in gestation (compare Fig. 6E and H).

**Figure 7:**
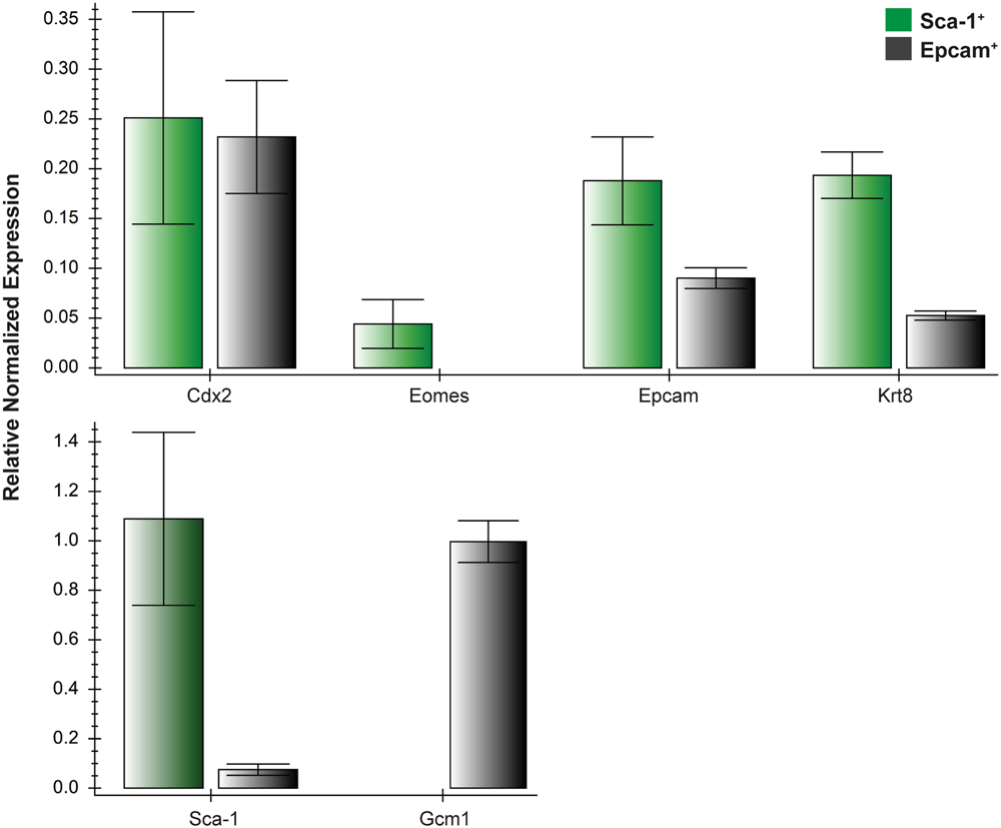
Sca-1^+^ and Epcam^+^ cells isolated from mouse placentae at E11.5 expressed the trophoblast marker, *Krt8* by qRT-PCR. These cells also expressed other known markers of trophoblast, including *Cdx2, Eomes* and *Epcam*. *Epcam* was expressed in both Sca-1^+^ and Epcam^+^ cells however *Sca-1* was only detected in Sca-1^+^ cells. The syncytiotrophoblast marker, *Gcm1*, was detected only in Epcam^+^ cells.

To assess whether Sca-1^+^ trophoblast cells could be successfully isolated from the placenta, we attempted isolation of both Sca-1^+^ and Epcam^+^ (as control) populations from E11.5 placentae. We chose this gestational age for several reasons: 1) the placenta is still growing and actively-dividing trophoblast cells are present^18^, thereby increasing our probability of isolating a proliferative cell population, 2) TS cells are thought to be depleted by this time in gestation^1^ and therefore, isolation of a proliferative trophoblast cell population would be novel, 3) previously reported trophoblast progenitor cells (SM10 and Epcam^+^) were isolated at E10.5 and we wanted to investigate a gestational age beyond this to support our hypothesis^18, 30^. Isolation of Sca-1^+^ and Epcam^+^ trophoblast was successful as shown by qRT-PCR analysis. Both Sca-1^+^ and Epcam^+^ cells expressed the trophoblast marker, *keratin 8* (*Krt8*), as well as *Cdx2* and *Epcam*. *Sca-1* was also detected in Epcam^+^ cells, though at a very low level and *Eomes* was only expressed in Sca-1^+^ cells, while Epcam^+^ cells strongly expressed *Gcm1* (Fig. 7), confirming that sorting isolated independent Sca-1^+^ and Epcam^+^ trophoblast populations from the placenta.

### Characterization of placental Sca-1^+^ trophoblast cells

**Figure 8:**
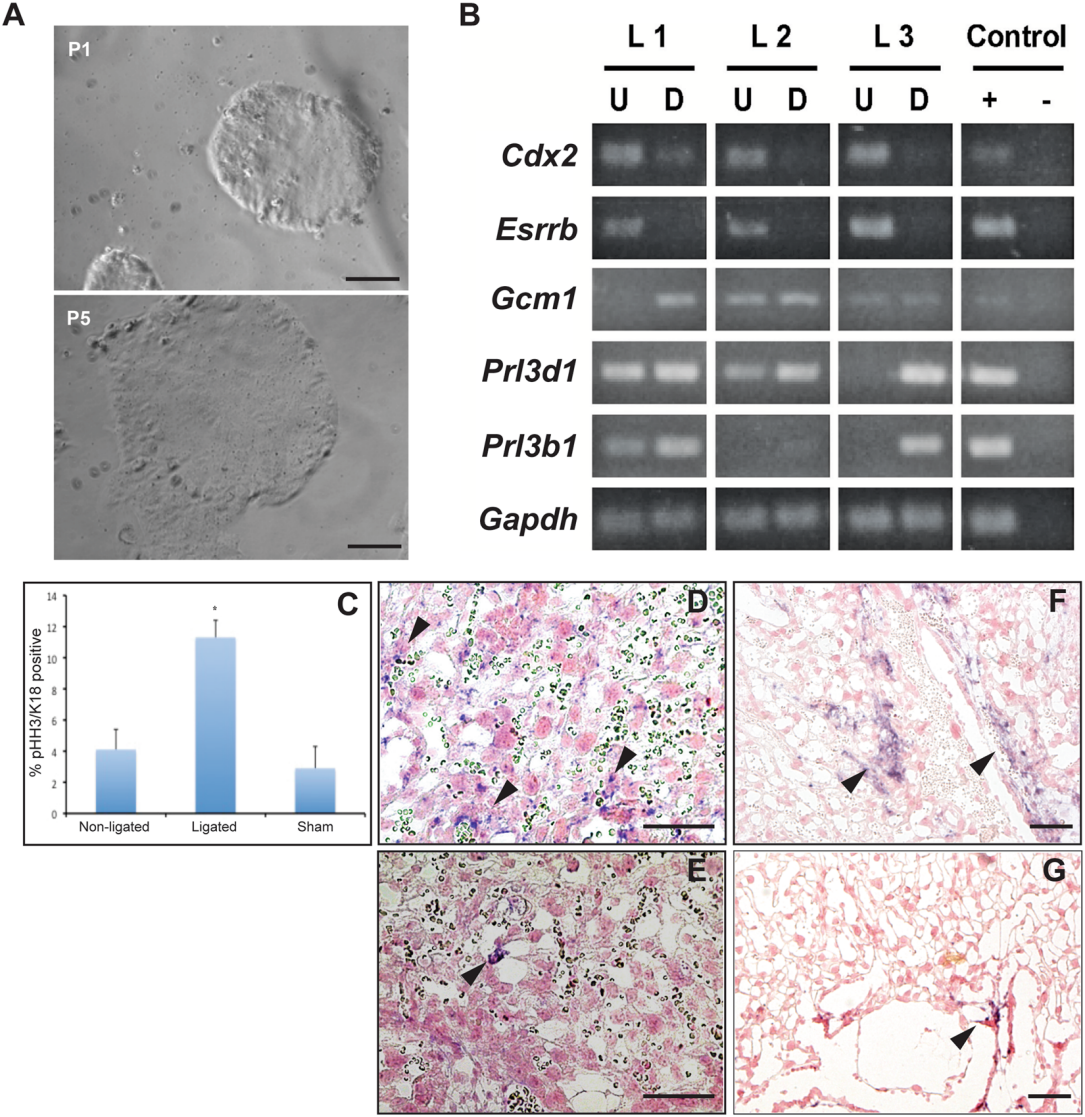
Sca-1^+^ cells isolated from E11.5 placentae formed proliferating, epithelial-like colonies that could be repeatedly passaged (**A**). When analyzed by RT-PCR, three independent Sca-1-derived colonies expressed genes indicative of trophoblast stem cells (*Cdx2, Esrrb*), syncytiotrophoblast (*Gcm1*) and trophoblast giant cells (*Prl3d1, Prl3b1*) (**B**). The frequency of pHH3^+^/cytokeratin^+^ trophoblast was increased in the labyrinth layer in response to RUPP (**C**). The expression patterns of *Eomes* (**D**) and *Sca-1* (**F**) were expanded in RUPP placentae (**D,F**) when compared to sham-operated controls (**E**, *Eomes*; **G**, *Sca-1*). U and D reflect undifferentiated and differentiated cultures respectively. Scale bar = 100 um. RT-PCR results presented in (**B**) are cropped and organized to be shown as a composite from multiple gel pictures.

Having confirmed that Sca-1 was an effective cell surface marker for the identification and isolation of a subpopulation of trophoblast cells from the placenta, it was important to evaluate whether their proliferation and differentiation potential was the same as those sorted from TS cell culture. Sca-1^+^ cells comprised ~4% of the total single-cell suspension, however, it should be noted that Sca-1 is also expressed in hematopoietic stem cells and neither MACS nor FACS directly distinguishes between the trophoblast or hematopoietic lineage; as such it was essential that at all stages, cells were assessed to confirm trophoblast lineage. As TS cell media and culture conditions preferentially promote trophoblast growth, placenta-isolated Sca-1^+^ cells were immediately placed in TS cell culture supplemented with growth factors to support formation of proliferative trophoblast colonies. Cells were plated, and 6 hours later, media and all non-attached cells were aspirated, and fresh media was added. Sca-1^**−**^ cells were plated as controls. To avoid disrupting the newly adhered cells and prevent differentiating or killing them by removing and counting them, colonies were calculated relative to the total number of cells plated, rather than the number of adherent cells. Within 5 days of the sort, Sca-1^+^ cells had new colonies forming while Sca-1^−^ cells had no colonies. Colonies were observed at a frequency of approximately 0.6 % (+/−0.05 %) of the total Sca-1^+^ cells plated and trophoblast lineage was confirmed by qRT-PCR. Once colonies had formed, the growth pattern was similar to established TS cell lines and TS cell-isolated Sca-1^+^ populations. Colonies were composed of small, tightly packed cells with borders that were smooth and resembled those formed by established TS cell lines (Fig. 8A). These cells remained proliferative and could be maintained and passaged indefinitely (at least 45 passages).

The potential of the placenta-isolated Sca-1^+^ trophoblast cells in both proliferating and differentiating conditions was assessed by RT-PCR. Three independently isolated Sca-1^+^ cell lines were analyzed in the presence of growth factors; all expressed *Cdx2* and *Esrrb* (Fig. 8B). As with TS cell cultures, removal of growth factors, stimulated differentiation of these Sca-1-derived colonies. Down regulation of *Cdx2* and *Esrrb* was associated with an increase in markers of differentiated trophoblast cells of the labyrinth and the junctional zone, including *Gcm1* (syncytiotrophoblast), *Prl3d1* (P-TGCs; also known as Pl1) and *Prl3b1* (P-TGCs and S-TGCs; also known as Pl2), (Fig. 8B), showing that placenta-isolated Sca-1^+^ trophoblast cells express markers of multiple trophoblast lineages, of both layers.

### Increased trophoblast proliferation in response to Reduced uteroplacental perfusion pressure (RUPP)

The RUPP model is a well-established model of placental insufficiency, IUGR and preeclampsia, involving a physical reduction in the blood supply to the uterus and therefore the fetoplacental units by surgically ligating maternal uterine arteries. Arteries are ligated just distal to the ovaries and on both uterine horns to reduce blood flow to the placenta. This model was established in the rat and has been applied in the mouse as a reproducible model of placental insufficiency and IUGR^31–34^. We applied RUPP at E14.5, a time when the mouse placenta is considered completely developed. Histological analysis of RUPP-IUGR placentae showed an increase in pHH3 staining when compared to sham operated controls. At E16.5, a 3.6 fold increase in the number of pHH3^+^/cytokeratin^+^ cells (Fig. 8C) corresponded with increased labyrinth-specific *Eomes* (compare Fig. 8D,E), and *Sca-1* expression in the RUPP vs. sham-operated placentae (compare Fig. 8F,G).

### Hypoxia affects Sca-1^+^ trophoblast potential

**Figure 9:**
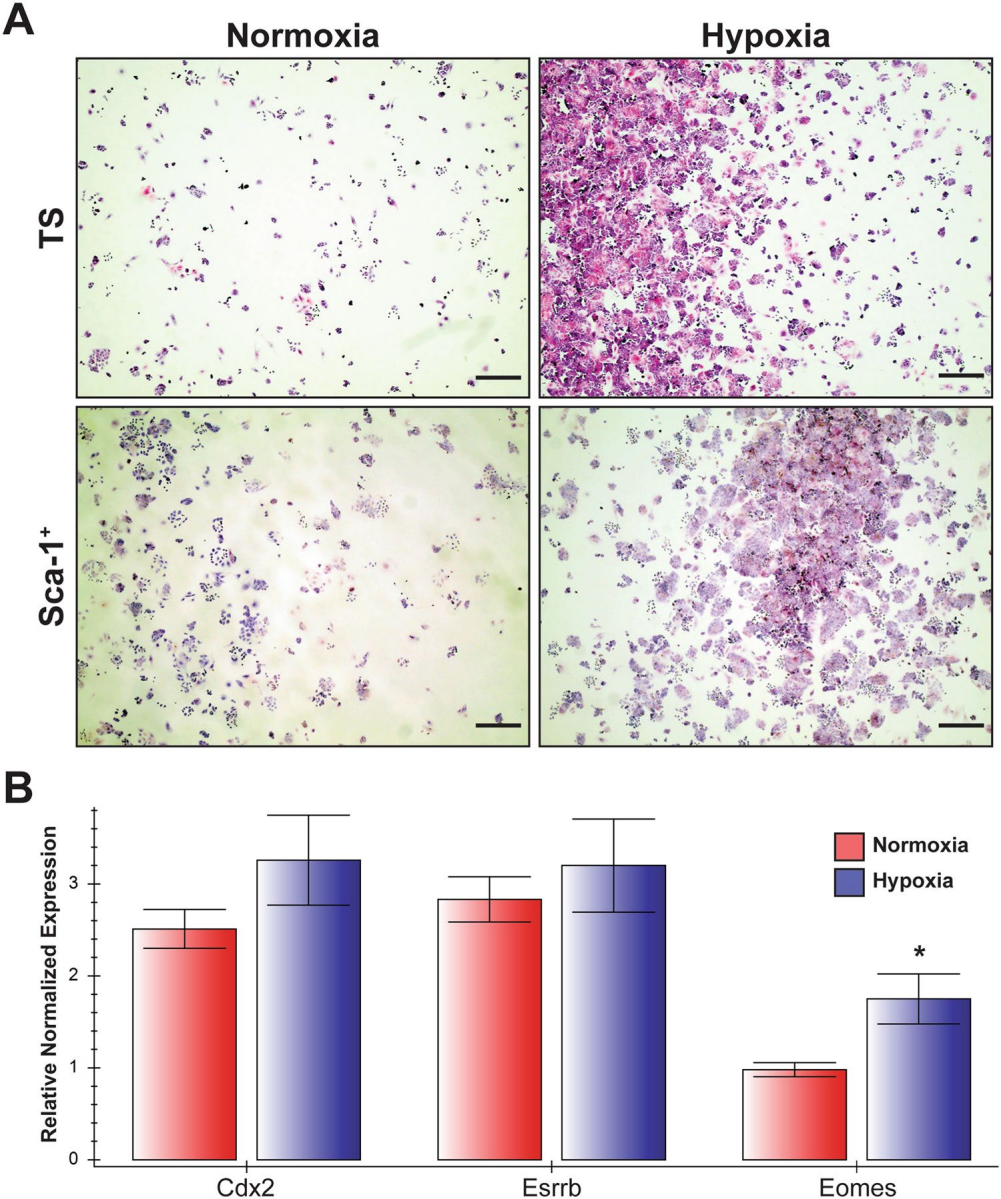
Sca-1^+^ trophoblast isolated from differentiated TS cell cultures were re-plated in TS media plus growth factors and cultured in 5% CO_2_ in air (Normoxia) or 1% O_2_ (Hypoxia) for 48 hours. These cultures were compared to proliferating, non-sorted TS cells plated in the same conditions and at the same seeding density (TS). Hypoxia promoted colony formation and proliferation compared to normoxia (**A**). After 48 hours of culture in hypoxia in the presence of growth factors, expression of *Cdx2* and *Esrrb* in Sca-1^+^ trophoblast was maintained, while *Eomes* expression was up regulated in these cells as detected by qRT-PCR (**B**). Cells were stained with hematoxylin and eosin. Scale bar = 500 um.

Increased labyrinth-specific *Sca-1* ^+^ expression and trophoblast proliferation in RUPP placentae, led us to question whether these changes could be in response to hypoxia. *In vitro* hypoxic studies used MACS-isolated Sca-1^+^ trophoblast subpopulation from TS cell cultures. TS and Sca-1^+^ cells were subjected to prolonged hypoxic culture for 6 days (1% oxygen) and compared to replicate controls cultured in normoxic conditions (standard tissue culture, 5% CO_2_ in air). TS cells and Sca-1^+^ subpopulations formed colonies and proliferated at a greater rate in low oxygen (Fig. 9A). We noted that both populations formed colonies in the center of the plates that started to over-grow before the colonies towards the edges became confluent, while in normoxia, cell growth was even across the plates (Fig. 9A). After 48 hours in hypoxia, Sca-1^+^ cells maintained expression of *Cdx2* and *Esrrb*, and *Eomes* expression was higher when compared to normoxia (Fig. 9B), thereby implicating a role for hypoxia in maintenance of stem cell marker expression and control of their proliferative nature.

## Discussion

TS cell lines derived from the mouse blastocyst are identifiable *in vitro* by the expression of transcription factors Cdx2, Esrrb and Eomes^27^. *In vivo* these same markers identify TS cells until E8.5, however isolating this population has been challenging, relying on micro-dissection of ExE/chorion^1^. Furthermore, while lineage-specific, proliferative progenitors have been identified in both the junctional zone^14, 15^ and labyrinth layer^18^, there has been no description of a population of trophoblast cells that persists beyond mid-gestation and is capable of proliferating and differentiating to all trophoblast subtypes.

This study identified Sca-1 as a cell-surface-marker of a population of trophoblast cells with proliferative potential. Sca-1 expression analysis, showed that both the mRNA and protein were highly expressed in undifferentiated TS cell cultures and when differentiated, expression was quickly down-regulated, which matches that of *Cdx2, Eomes* and *Esrrb*, markers of TS cells. Because the Sca-1 null embryos are viable, we did not explore the functional role of Sca-1 in this study. However, we did consider the possibility that Sca-1 expression may simply be a reflection of FGF4 exposure. However, while removal of FGF4 reduced Sca-1 expression, there remained a small subpopulation of Sca-1^+^ cells, that when returned to FGF4^+^ media, saw an increase in Sca-1 and other TS cell markers and was associated with colony-forming ability. Because Sca-1 expression, albeit low, is maintained in this population, we do not believe that it is a direct target of FGF4, though speculate that it may be related to the Esrrb pathway as Sca-1^+^ cells showed increased *Esrrb* expression over their non-sorted, TS cell counterparts and Esrrb is a downstream target of fibroblast grown factor (FGF) signaling and is critical to drive TS cell self-renewal^35^. We propose that Sca-1 is a good cell-surface-marker of a population of trophoblast with proliferative potential and to our knowledge, a persistent proliferative trophoblast subpopulation among terminally differentiated trophoblast cultures has not been previously identified.

Before addressing the colony-forming potential of Sca-1^+^ cells, it was important to establish a baseline for TS cells. We found that TS cells from undifferentiated cultures formed proliferating colonies at a frequency of ~1/15, which was not unexpected as TS cultures are not homogenous in their “stemness”, as previously discussed. This is also noted within other stem cell populations in culture, that not every cell will form colonies^36^. The Sca-1^+^ population isolated from differentiated TS cell cultures had colony-forming frequency of ~1/42, which is approximately 1/3 that of the TS cells from proliferating cultures. The possibility that it was the sort itself that reduced the colony forming frequency of the Sca-1^+^ population was considered but cells from proliferating cultures were also FAC sorted. Several factors may also influence colony-forming ability, including both cell-cell interactions and paracrine signaling. With respect to paracrine signaling, it is possible that once some of the cells begin to proliferate and form colonies, they produce paracrine factors, which then signal to the surrounding cells to stop proliferating and/or to differentiate. Such interactions are observed in other stem cell niches; for example, the bulge stem cells of the hair follicle are maintained in a quiescent state through paracrine signaling from other cells within the niche (reviewed in Hsu *et al*.^37^). Of note, no colonies were formed in the Sca-1-depleted population, highlighting that the cells with persistent, proliferative potential were in fact Sca-1^+^. Whether every Sca-1^+^ cell has the potential to be colony forming and proliferate is still unknown however, our findings allow us to conclude that the colony-forming, proliferative population among differentiated trophoblast cells, expresses Sca-1. We propose that understanding how these factors influence this population’s ability to persist and proliferate will be key to understanding the role of these cells.

**Figure 10:**
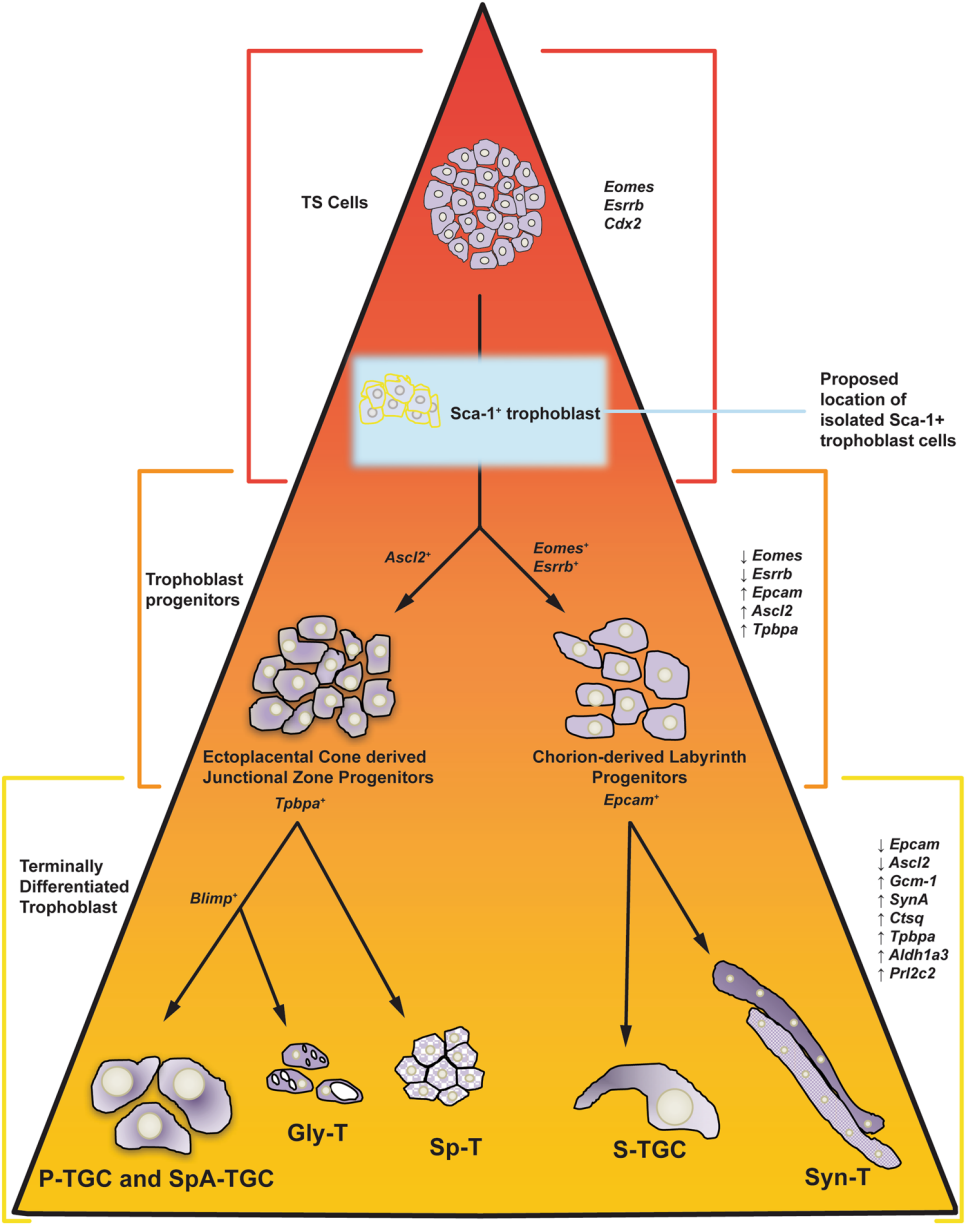
Cartoon depicting proposed position of Sca-1^+^ cells within the mouse trophoblast stem cell lineage. P-TGC, parietal trophoblast giant cell; SpA-TGC, spiral artery-associated trophoblast giant cell; Gly-T, glycogen trophoblast; Sp-T, spongiotrophoblast; S-TGC, sinusoidal trophoblast giant cell; Syn-T, syncytiotrophoblast.

We showed that Sca-1^+^-derived colonies cells could proliferate over many passages and established stable cell lines, thereby demonstrating their proliferative potential. Together with the fact that the Sca-1^**−**^ trophoblast population did not proliferate, and that the Sca-1^+^ subpopulation had increased levels of the TS markers, *Cdx2*, and *Esrrb* when compared to undifferentiated TS cell cultures, suggests that selecting based on Sca-1 expression isolates a subpopulation with a more homogeneous “stemness”. While Sca-1^+^ cells expressed *Epcam* mRNA, Epcam^+^ (labyrinth progenitor) cells only expressed the message for *Sca-1* at very low levels. This suggests that TS cells sorted based on the expression of the cell surface protein-Sca-1 have the potential to differentiate to Epcam^+^ progenitors, as indicated by the mRNA expression of *Epcam*, while the TS cells sorted based on the expression of the cell surface protein-Epcam have already progressed in their differentiation lineage to the point of trophoblast progenitor, as indicated by the absence and/or very low levels of TS cell markers and *Sca-1* (See Fig. 10 for proposed trophoblast lineage). Furthermore, the cell lines derived from Sca-1^+^ colonies could be differentiated into all trophoblast sub-types, including progenitors and terminally differentiated trophoblast of the junctional zone and the labyrinth. We speculate that the Sca-1^+^ subpopulation is closer to a stem cell-like population than a layer-restricted progenitor population in that they have multipotent potential. We have shown that *in vitro* Sca-1 expression identifies a persistent, undifferentiated trophoblast population with proliferative, colony-forming potential, which under permissive conditions can differentiate to trophoblast subtypes of both the labyrinth and the junctional zone.

It has been suggested that there is not a stem cell nor multi-potent progenitor trophoblast population in the placenta beyond E8.5 and E14.5, respectively. Our data showed that Sca-1 was expressed in the chorion and labyrinth layer of E8.5-E18.5 placenta, albeit with decreasing frequency as gestation continued. Though Sca-1-driven EGFP was observed in the labyrinth layer by Ottersbach *et al*., was suggested to be expressed in the trophoblast, these cells had never been characterized^22^. We showed that FACS and/or MACS allowed for successful isolation of Sca-1^+^ trophoblast cells from culture, therefore we sought to isolate and characterize these cells *in vivo*.

Sca-1 expression in trophoblast cells was restricted to the labyrinth layer and overlapped with Epcam expression at early stages of labyrinth development. However, similarly to our TS cell culture experiments, Sca-1^+^ and Epcam^+^ populations appeared to be different populations later in development and had different expression of trophoblast markers when isolated from the E11.5 placenta, confirming that these populations were distinct *in vivo*. The placenta-isolated Sca-1^+^ cells were cultured and analysis confirmed that they were of trophoblast lineage with the same proliferation and differentiation potential as the Sca-1^+^ cells isolated from TS cell culture. As with TS cell cultures, removal of growth factors stimulated differentiation of the Sca-1-derived colonies showing that they too were FGF4 responsive. In response, TS cell markers were down regulated and the markers of terminally differentiated trophoblast cells of both the labyrinth and the junctional zone were detected. Our results demonstrated that the placenta-derived Sca-1^+^ cell lines expressed markers of both trophoblast stem cells and differentiated trophoblast subtypes when cultured under proliferating and differentiating conditions respectively, showing that is it possible to use Sca-1 as a cell surface marker to isolate a persistent population with proliferative, multipotent trophoblast stem cell-like potential from the placenta.

The colony formation of the placenta-derived Sca-1^+^ population of ~0.6%, is approximately one quarter of that suggested by our limiting dilution studies on TS cell-isolated Sca-1^+^ cells from differentiating cultures (~2.4%). To avoid compromising the sorted cells by recounting after plating, our numbers reflect the frequency of colonies formed from all Sca-1^+^ cells isolated, rather than just the adhered Sca-1^+^ trophoblast, as such many non-adherent cells were removed from the culture after 6 hours when the media was aspirated and therefore, the numbers may be biased; accounting for the lower frequency of colony-formation from Sca-1^+^ cells from placenta versus TS cell culture. We acknowledge that the lower number of colonies may also reflect the stress of isolation from the placenta as well as the fact that the cells have been removed from their niche in the placenta, thereby promoting differentiation. Even under optimal cell culture conditions that include FGF4 and embryonic fetal fibroblast feeders, a proportion of cells in established TS cell cultures spontaneously undergo differentiation^27^. In similar experiments to sort Sca-1^+^ cardiac progenitor cells from mouse adult hearts, Sca-1^+^ cells formed beating cardiomyocytes, but only at a rate of 1% of total Sca-1^+^ cells isolated^38^. In our experiments, while the isolation may yield small numbers of colony-forming cells, it is notable that proliferating cell lines could be derived. It is tempting to speculate that optimization of tissue culture media or addition of extracellular matrix might enhance the success of colony formation. For example, Ohinata *et al*., showed that the frequency of deriving stable TS cell lines from blastocysts could be increased by removing serum and culturing on fibronectin, using a completely defined culture media which included FGF2, Activin A and the canonical Wnt and Rho-associated protein kinase inhibitors XAV939 and Y27632, respectively^23^.

Addressing the functional role of Sca-1 is an interesting question for future studies. However, it has been published that targeted mutation of Sca-1 does not result in any developmental phenotypes in homozygous null pups^39^, suggesting that Sca-1 itself, may not be crucial for normal trophoblast stem cell maintenance. However, hematopoietic stem cells isolated from Sca-1 homozygous null animals are deficient in long-term repopulation of the HSC niche following bone marrow transplant to lethally irradiated recipients^40^, suggesting that Sca-1 may function in the long-term maintenance of stem cells or that Sca-1 may play a functional role in response to stress. In the placenta, a phenotype resulting from Sca-1 mutation may become apparent in situations of stress during pregnancy, for example where there is placental insufficiency. Further investigation into the role of Sca-1 in the TS cell niche will be required to fully understand its role, however, the use of the gene as a cell surface marker to isolate persistent, proliferative multipotent trophoblast is of value.

In response to RUPP, we observed an increase in the frequency of pHH3^+^ trophoblast cells, which corresponded with increased labyrinth-specific *Eomes* and *Sca-1* expression. A response to stress and/or injury by tissue-specific stem/progenitor cells has been described in other organs^2, 3^. Increases in proliferating trophoblast cells have been observed in placentae from human pregnancies affected by pre-eclampsia^41^.

Using RUPP to model preeclampsia in the mouse, Fushima *et al*., showed that this model resulted in placental hypoxia, leading to increased Hif-1a expression^34^. Hypoxia is a known regulator of cell proliferation and differentiation wherein it can result in either proliferation or cell cycle exit, depending on the cell type^42^. Hypothesizing that it could be hypoxic stress that caused the increased expression of *Eomes* and *Sca-1* in our RUPP experiments, we showed that proliferation of TS- and Sca-1^+^ cells was increased in response to hypoxia *in vitro*. Both populations of cells became confluent in a shorter time and markers of TS cells were maintained or increased, suggesting that hypoxia stimulated both proliferation and maintenance of undifferentiated cells. Additionally, in the TS cell population we observed increased *Sca-1* expression in response to hypoxia. Proliferation and differentiation of Sca-1^+^ trophoblast cells was altered by hypoxia and the *in vivo*/*in vitro* data together suggest that hypoxia may be responsible for the increased proliferation and simultaneous increase in labyrinth associated *Eomes/Sca-1* expression in the RUPP placentae when compared to their sham counterparts. Our lab is currently focusing on additional experiments using lineage-tracing approaches to further evaluate the contribution and potential of Sca-1^+^ trophoblast cells in this model.

Previous studies showed that Epcam^+^ labyrinth progenitor cells persist in the placenta until E14.5 to support growth and development. Our analysis suggests that this progenitor population may persist until E18.5; but is distinct from the subpopulation of Sca-1^+^ trophoblast cells that we have identified. We show that 1) Sca-1 can be used as a mouse cell surface marker to identify and isolate *in vitro* and *in vivo* trophoblast subpopulations with colony forming potential, 2) these placenta- and TS cell-isolated Sca-1^+^ trophoblast cells can differentiate *in vitro* to trophoblast subtypes found in both layers of the mature placenta and 3) there is a relationship between *Sca-1* expression/proliferation and hypoxic stress. A human homolog of Sca-1 has not yet been identified. However, we anticipate that further characterization of mouse Sca-1^+^ trophoblast and examination of global gene expression in these cells when compared to human trophoblast could lead to the identification of genes, common to the mouse and human trophoblast. In turn, this should move us closer to the identification, isolation and understanding of trophoblast stem cells in the human placenta.

## Materials and Methods

### Animals and cell culture

Placentae were dissected at different stages of gestation from pregnant CD-1 females. Placentae were fixed in 4% paraformaldehyde overnight, embedded in paraffin and sectioned for analysis by histological staining; immunohistochemistry and *in situ* hybridization as previously described^17, 26, 43^. RUPP experiment surgeries were conducted as previously described in the rat^32^ and the mouse^34^ but modified to include bilateral ligation of the uterine arteries with surgical thread instead of a surgical clip. Sham and non-operated controls, in which no ligation and no surgery were performed respectively, were used as controls. Isolation of trophoblast single cell suspensions was conducted as previously described^44^ and modified to include the use of a Percoll gradient^45^. All mouse work was conducted in accordance with an animal protocol assessed and approved by the University of Calgary, Animal Care Committee under guidelines for use of animal models in research as outlined in “Guide to the Care and Use of Experimental Animals” by the Canadian Council on Animal Care.

Primary trophoblast cells and established TS cell lines were cultured in 5% CO_2_ in humidified air at 37°C in RPMI culture media supplemented with 20% FBS, 1 mM sodium pyruvate, 50 μg/mL penicillin/streptomycin, 25 ng/mL FGF4, 10 ng/mL recombinant human Activin A, 1 μg/mL heparin and 5.5 × 10^−5^ M β-mercaptoethanol as previously described to maintain undifferentiated, proliferating cells^26^. Cells were stimulated to differentiate by the removal of FGF4, activin and heparin.

### Immunostaining, Fluorescence Activated Cell Sorting (FACS) and Magnetic Activated Cells Sorting (MACS) trophoblast cell isolation

**I**mmuno**h**isto**c**hemistry (IHC), to identify the expression of Sca-1, Epcam and phosphor-histone H3 (pHH3) and **i**mmuno**f**luorescence (IF), to identify Sca-1 and pHH3 in the placenta, were conducted on 5–7 μm tissue sections. Sections were de-paraffinized and rehydrated followed by antigen retrieval in Citra Buffer (Biogenex, USA) using a 2100-Retriever (Electron Microscopy Science, USA). The IHC sections were then treated with 3% H_2_0_2_ to quench endogenous peroxidase, washed in PBS and blocked for 1 hour at room temperature in 1x PBS, 5% goat serum, 0.1% BSA. Sections were incubated at room temperature for 1 hour in anti-Sca-1 (Abcam Cat# ab109211, Abcam; 557403, clone D7, RRID:AB_10862573; BD Biosciences; Cat# 557403, RRID: AB_396686; Abcam Cat# 3121-1, Epitomics RRID:AB_2234743) or anti-Epcam antibody (Abcam Cat# ab124825, RRID:AB_10973714), diluted 1:100 in block solution. The anti-pHH3 (Millipore Cat# 06-570, EMD Millipore, RRID:AB_310177), antibody was diluted 1:300 in block solution. The remainder of the protocol followed the manufacturer’s protocol (Vectastain, Vector Labs, USA). Antibody binding was visualized using Dako DAB according to the manufacturer’s protocol (Dako, USA). Following counterstaining in hematoxylin (Gills #2, Sigma, USA), sections were dehydrated and mounted in xylene-based mounting medium. The IF sections were de-paraffinized, underwent antigen retrieval and were blocked as for IHC. Sections were then exposed to anti-pHH3 (Millipore Cat# 06-570, EMD Millipore, RRID:AB_310177) and anti-cytokeratin (Dako Cat# Z0622; DakoCytomation, RRID:AB_2650434), diluted 1:100 and 1:300, respectively in block and incubated overnight at 4°C. Following primary antibody incubation, sections were washed in PBS and exposed to Cy5-anti-rabbit and Alexa488-anti rat 2° antibodies (111-175-144, Jackson ImmunoResearch Labs Cat# 111-175-144, RRIB:AB_2338013 and A-11006, ThermoFisherThermo Fisher Scientific Cat# A-11006, RRID:AB_2534074, respectively), both diluted 1:300 in block, for 1 hour at room temperature followed by PBS wash, counterstaining of nuclei with Hoechst 33342 (1:1000, Molecular Probes, Invitrogen, USA) as previously described^46^.

Sca-1^+^ trophoblast cells were identified in TS cell cultures or in single cell suspensions from dissected placentae by FACS as previously described^46^. Placentae were dissected to isolate the labyrinth/junctional zone from the maternal decidua and single cell suspensions were then generated. The isolation of the Sca-1^+^ subpopulation of cells used FACS^46^ or commercially available microbead isolation protocols (according to manufacturer; Miltenyi Biotec). Anti-Sca-1 antibodies were from BD Biosciences (557403, clone D7) and Miltenyi (130-102-297, clone D7). The isolation of Epcam^+^ cells from differentiated TS cell cultures, used a commercially available microbead isolation protocol, and was also used with commercially available antibodies (CD326, 130-102-214, clone caa7-9GB, Miltenyi Biotec).

*In vitro* studies using TS cells were cultured as previously described^26^. Limiting dilution assays were carried out by plating 100, 50, 10, 5 or 1 cell(s) per each well of a 96-well plate for each population of trophoblast cells examined in an experiment. Each experiment was conducted three times and the results were tabulated and assessed using the ELDA (Extreme Limiting Dilution Analysis) tool^28^.

### Hypoxia Chamber

Inflatable hypoxic chambers were adapted and modified from a published study^47^, using sterile 4.5 mm heat-sealable pouches (VWR, USA). Briefly, culture dishes were placed inside pouches and the open end was heat-sealed to close. Two corners were then cut on a 45° angle just large enough to allow a 5 mL serological pipette fitted to flexible tubing (and attached to mixed air tank) to be inserted in one corner. The second corner was left open to allow air to exit the pouch as it was flushed with 1% oxygen mix. Pouches were flushed with 1% mixed air for approximately 3 minutes at which point the open corner was heat sealed. The pouch was then inflated to ~80% capacity at which point the remaining open corner was sealed and the chamber was placed in the tissue culture incubator. Individual chambers were made to allow for collection of cells after 2, 4, and 6 days and filled with mixed air containing 1% oxygen, 5% CO_2_, and N_2_ balanced (Airgas, USA). Cells were collected for RNA in RNA lysis buffer (Aurum, BioRad, USA), or were fixed in 4% paraformaldehyde (Sigma, USA), for 20 minutes at room temperature.

### Histological Staining of Cultured Cells

Following fixation, cells were washed and assessed by standard **H**ematoxylin and **E**osin (H&E) Stain (according to manufacturer’s protocol; Sigma, USA). **P**eriodic **A**cid-**S**chiff (PAS) staining was used to assess for glycogen accumulation within the cells using the PAS kit (395B-1KT, Sigma-Aldrich) without pre-digestion with diastase. **A**lkaline **P**hosphatase (AP) staining was used to assess for differentiation of sinusoidal trophoblast giant cells. Briefly, cells were rinsed in PBS, incubated in wash buffer (pH 9.5) and alkaline phosphatase activity was detected by using NBT/BCIP substrate to form a blue precipitate. The reaction was stopped in PBS and cells were counterstained in Nuclear Fast Red (Vector Labs, USA) for 1 min, followed by dehydration through a graded ethanol series. Images were collected using a Leica DMR light microscope (fluorescence and bright-field) and an EVOS XL Core microscope (bright-field; Invitrogen, USA).

### RNA isolation and analysis of gene expression

Total RNA was isolated from TS cell cultures using RNeasy or Aurum microspin columns according to the manufacturer’s instructions (Qiagen and BioRad, USA, respectively). Northern blots were conducted using 10 μg of total RNA as previously described^26^. Membranes were probed with ^32^P-labelled cDNA probes for Esrrb, Eomes, Gcm1, Syna, Tpbpa, Pl1 and Rn18s ribosomal RNA as previously published^26^. A Sca-1 cDNA probe was generated by PCR (see below).

mRNA expression was assessed by quantitative **r**everse **t**ranscription (RT)-PCR using the SYBR green method as previously described^46, 48^. 1 μg of total RNA was reverse transcribed using the iScript RT kit for SYBR green (BioRad, USA). qRT-PCR reactions were then prepared using the iTaq Universal SYBR green supermix (BioRad, USA), according to the manufacturer’s instructions, and performed on a BioRad CFX96 thermocycler. Primers for Sca-1(Ly6a; qMmuCED0003761), Esrrb (qMmuCED0039638), Epcam (qMmuCID0017186), Rhox4 (qMmuCID0041300), Gcm1 (qMmuCID0023712), Syna (qMmuCed0003216), Tpbpa (qMmuCID0007168), Pl1 (Prl3d1; qMmuCID0041026)), Plf (Prl2c2; qMmuCID0061688) and Ctsq (qMmuCID0022572) were ordered from BioRad (USA). qRT-PCR reactions were conducted in triplicate on cDNA representing three independent experimental replicates for each gene. Data was compiled and analyzed for significant changes in gene expression using the BioRad CFX Manager software, which utilizes the ΔΔCT method of analysis and using Ppia and Ywhaz (qMmuCED0041303 and qMmuCED0027504, respectively, BioRad) as reference genes^49^.

RNA $\underline{{\boldsymbol{i}}}n\,\underline{{\boldsymbol{s}}}itu$ ***h*** *ybridization* (ISH) was conducted on 7 μm sections of mouse placenta tissue as previously described^17, 26, 50^. *Gcm1* and *Prl3b1* cRNA probes are published^14, 17^, while *Sca-1* and *Epcam* probes were transcribed from PCR template generated using primers incorporating T7 and T3 RNA polymerase promoter sequences (Sca-1: Forward**/T3: 5′-AATTAACCCTCACTAAAGGG**CCATCAATTACCTGCCCCTA; Reverse/**T7: 5′-TAATACGACTCACTATAGGG**CTTCACTGTGCTGGCTGTGT; Epcam: Forward**/T3: 5′-AATTAACCCTCACTAAAGGG**CCTGAGAGTGAACGGAGAGC

Reverse**/T7: 5′-TAATACGACTCACTATAGGG**GGGCAGCCTTAATCACAAAA).
